# Case report: Rare pulmonary fungal infection caused by *Penicillium digitatum*: the first clinical report in China

**DOI:** 10.3389/fmed.2024.1424586

**Published:** 2024-07-17

**Authors:** Xiaojuan Shi, Jiaqing Ye, Peiling Liu, Weili Gao, Zhongjun Feng, Cuiying Zheng, Yinqi Huang, Yumei Guo, Lijie Zhang

**Affiliations:** ^1^Department of Respiratory and Critical Care Medicine, Hebei Medical University Third Hospital, Shijiazhuang, China; ^2^Department of Clinical Laboratory, Hebei Medical University Third Hospital, Shijiazhuang, China; ^3^Hebei Key Laboratory of Intractable Pathogens, Shijiazhuang Center for Disease Control and Prevention, Shijiazhuang, China

**Keywords:** *Penicillium digitatum*, fungal infection, mNGS, pulmonary infection, invasive pulmonary fungal infection

## Abstract

*Penicillium digitatum* is a common plant pathogen that causes citrus rot, which is extremely rare in humans. We report a case of a 66-year-old man with a history of consuming large amounts of citrus fruits, smoking for 30 years, and a history of emphysema. He had experienced intermittent coughing with sputum for more than 10 years and was admitted to the hospital due to worsening of symptoms over the past month. Despite antibiotic treatment, his condition did not improve. Subsequently, bronchoalveolar lavage fluid (BALF) was detected by metagenomic next-generation sequencing (mNGS), which showed the presence of *P. digitatum*. The fungal culture of BALF also indicated the presence of the *Penicillium* genus. The diagnosis was lung infection caused by *P. digitatum*, and the patient was treated with itraconazole. The lung infection was controlled. This is the third reported case of invasive pulmonary fungal infection caused by *P. digitatum* worldwide at the genus level, and the first reported case in China. Although human infections caused by *P. digitatum* are rare, as an emerging opportunistic pathogen, the detection of this fungus in immunocompromised patients should still be clinically important.

## Introduction

*Penicillium* species are among the most ubiquitous fungi in the environment (1) and rarely cause human infections. There have been only two reported cases of invasive pulmonary fungal infections caused by *Penicillium digitatum* at the genus level worldwide (2, 3). However, numerous reports suggest that *Penicillium spp*. can cause clinically significant diseases in immunocompromised individuals, such as urinary tract infections, corneal infections, cutaneous endocarditis, peritonitis, pneumonia, paravertebral infections, and brain infections (3–5). *Penicillium digitatum* belongs to the genus *Penicillium* and is one of the factors responsible for the decay of citrus fruits (6, 7). Although *P. digitatum* is a rare opportunistic pathogen causing human infections, we report a case of pulmonary fungal infection caused by *P. digitatum*, which is the first clinical case reported in China and the third reported worldwide.

## Case presentation

A 66-year-old man from Hebei, China, was admitted to the hospital on 4 February 2023 (day 1) due to a worsened cough and phlegm for 1 month. Despite the self-administration of cefuroxime, the symptoms did not improve. The patient had a chronic cough and expectoration for over 10 years. Two months ago, he suffered from pelvic fractures and underwent surgery. Six weeks ago, he was infected with COVID-19, which mainly manifested as a fever for 3 days, accompanied by pharyngalgia, cough, and sputum, without dyspnea. The patient visited a community hospital, where he was advised to isolate himself at home and rest without receiving glucocorticoids or other related treatments. His symptoms were basically under control. The patient had been smoking and drinking for over 30 years but had quit both 2 months prior to the visit.

**Figure 1:**
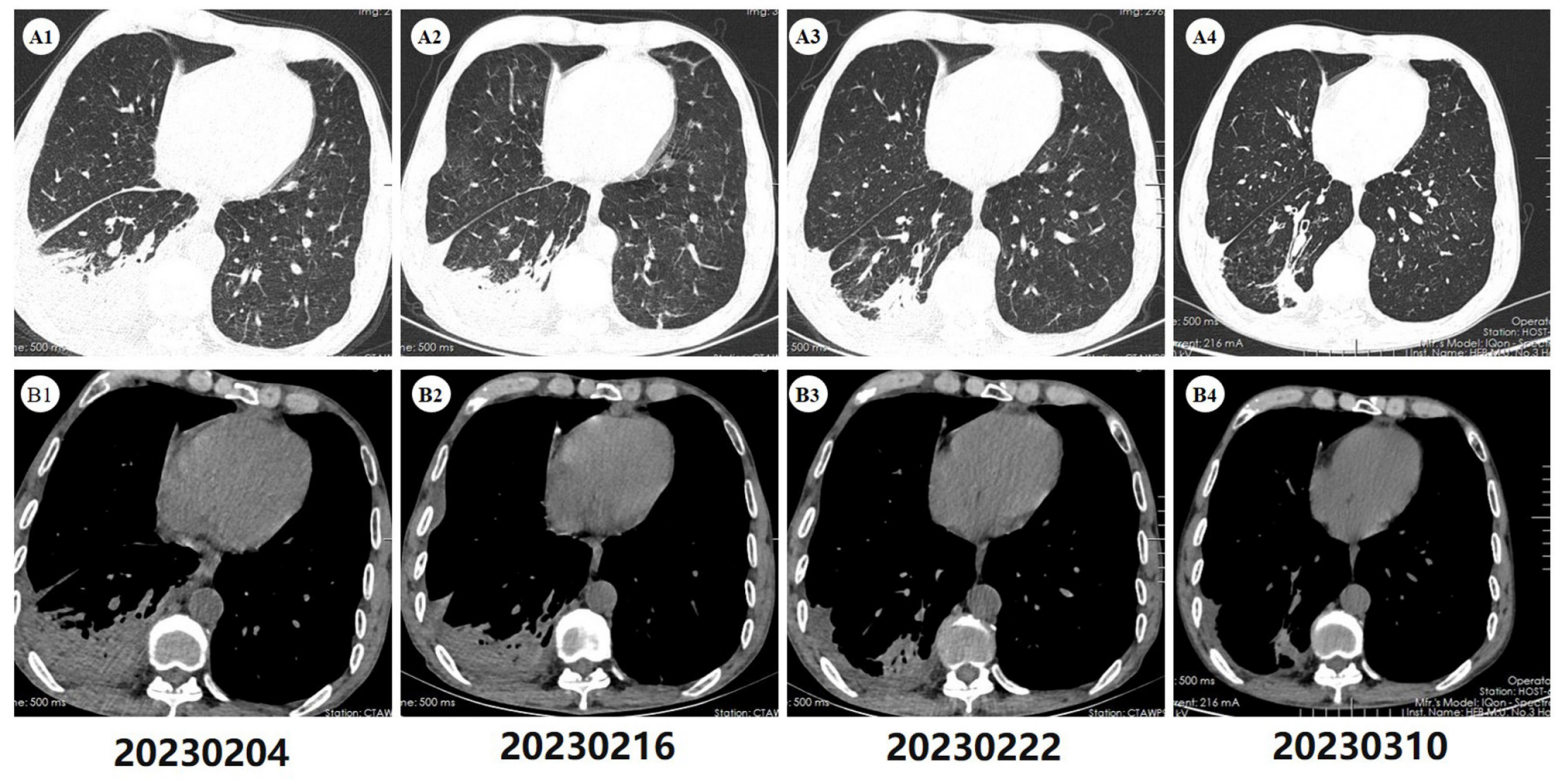
Computed tomography of the chest. **(A1, B1)** Upon admission, the scan (2023-02-04) revealed inflammatory lesions in the right lower lobe of the lung, along with localized consolidation. Furthermore, minimal pleural effusion was noted in the right pleural cavity and interlobar fissure. Bilateral pulmonary emphysema was also present. **(A2, B2)** A subsequent scan (2023-02-16), conducted after 12 days of antibiotic therapy, demonstrated persistent pulmonary lesions similar to those observed on the initial admission CT scan, with no apparent improvement. **(A3, B3)** A follow-up scan (2023-02-22), conducted after 6 days of antifungal therapy, exhibited improvement of pulmonary inflammation and pleural effusion compared to previous scans. **(A4, B4)** After 22 days of antifungal treatment, a follow-up scan (2023-03-10) demonstrated further absorption of the lung lesions, with only a small amount of residual fibrous streaky changes remaining.

On admission, the physical examination revealed that the patient was emaciated (body mass index = 16.53), with a barrel chest and low respiratory sounds in both lungs, especially in the right lower lung. A computed tomography (CT) scan revealed inflammatory lesions and local consolidation in the right lower lobe, with a small amount of pleural effusion on the right side and bilateral lung emphysema (Figures 1A1, B1). The laboratory results were as follows: WBC was 5.87 × 10^9^/L, but the lymphocyte count (LYM) was decreased to 0.7 × 10^9^/L. Additionally, the patient had mild anemia. The erythrocyte sedimentation rate (ESR) was significantly increased at 118.00 mm/h, and the high-sensitivity C-reactive protein (hsCRP) was also significantly elevated at 136.85 mg/L. Furthermore, the patient had significantly decreased levels of plasma albumin (ALB) at 26.63 g/L, while the liver and kidney function and arterial blood gas analysis indicators were essentially normal.

**Figure 2:**
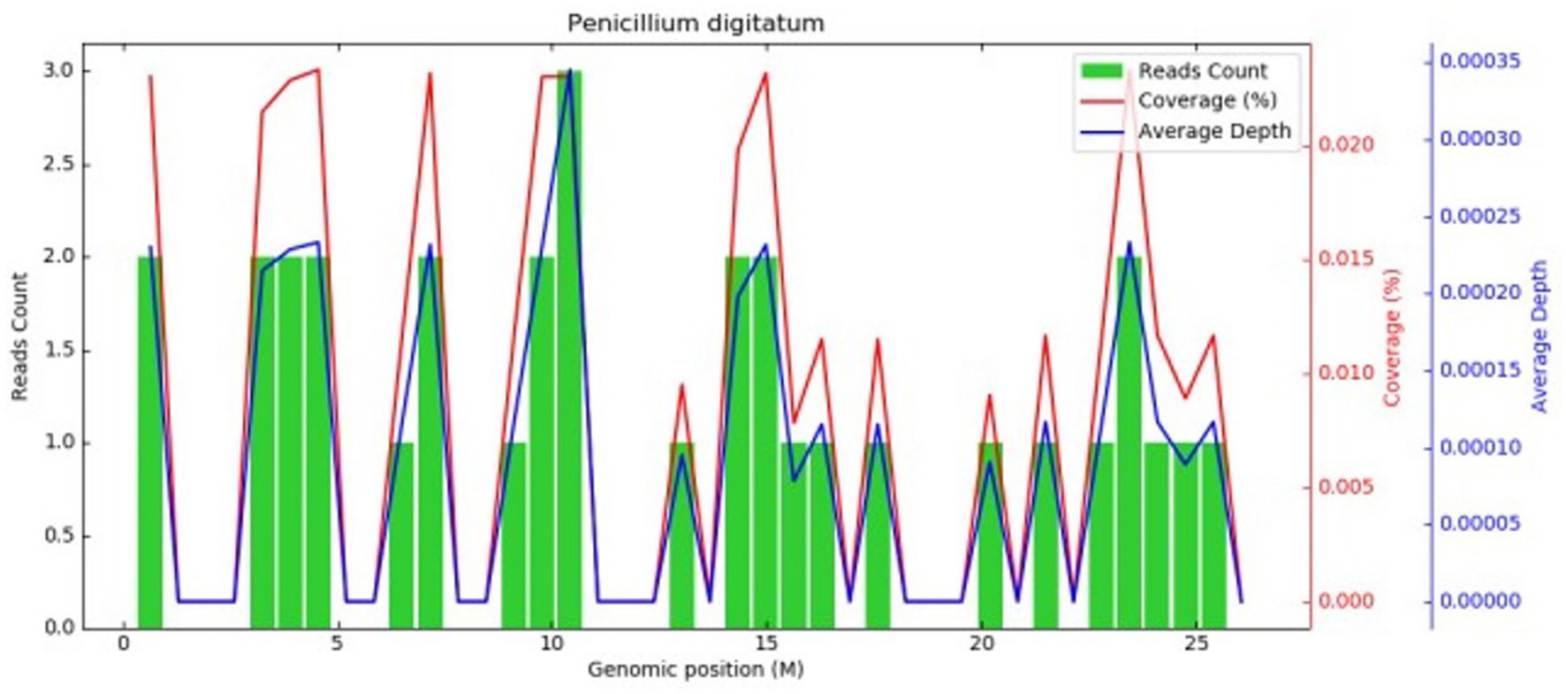
Metagenomic next-generation sequencing. Coverage graph of *P.digitatum* (36 reads, relative abundance value of 67.92%). The abscissa represents the genome position, the left ordinate is the number of reads of the pathogen aligned on the genome location (The number of map reads shown in green), and the right ordinate is the coverage and average sequencing depth at the corresponding position (coverage and depth, the red and blue lines in the figures).

**Figure 3:**
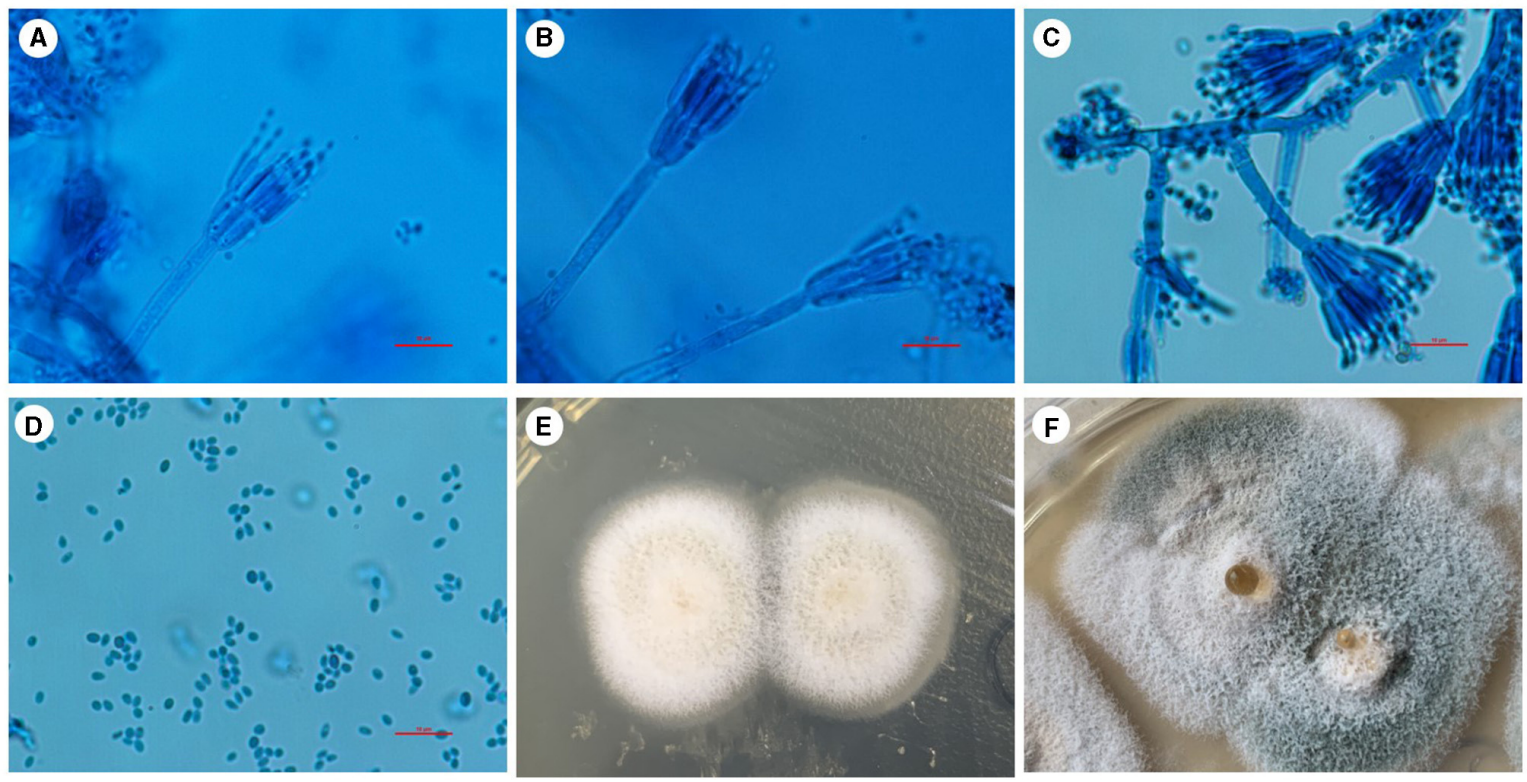
*Penicillium digitatum* was cultured for fungi in Sabouraud agar medium in a BALF sample at 28°C. **(A–C)** Positive colonies were cultured and stained with a lactophenol cotton blue staining solution. Under the microscope, the colonies showed a range of branching patterns, from simple to complex. The conidiophores had undergone multiple branchings and produced several rounds of symmetrical or asymmetrical small branches resembling brooms. **(D)** The spores of this *Penicillium* are typically elliptical under the microscope. **(E)** After 5 days of culture, the initial growth of *Penicillium* was observed, with white colonies exhibiting a velvety texture. **(F)** With the prolongation of growth time, the color of the colony gradually changes to light green or green, and liquid droplets appear in the center. Scale bars: 10 μm.

The initial diagnosis suggested that the patient might have community-acquired pneumonia, thus benzylpenicillin and levofloxacin were administered intravenously. After 7 days of treatment, the patient's symptoms did not improve, and repeated chest CT scans revealed no improvement in the pulmonary infiltrates or pleural effusion. The initial treatment was evaluated as ineffective, and three sputum smears, namely, sputum bacterial, fungal cultures, and acid-fast staining, were all negative. Therefore, the patient received an empirical upgrade of antimicrobial therapy with cefoperazone-sulbactam sodium and levofloxacin. Then, the bronchoalveolar lavage fluid (BALF) was collected and sent for metagenomic next-generation sequencing (mNGS), bacterial and fungal cultures, and acid-fast staining. The mNGS (SimcereDx, Nanjing, China) results were reported on the 2^nd^ day of sequencing (day 11), showing the presence of *Penicillium digitatum* (36 reads with a relative abundance value of 67.92%) (Figure 2). These sequences were submitted to the SRA database at NCBI with mNGS with the accession number PRJNA1102665 (https://dataview.ncbi.nlm.nih.gov/object/PRJNA1102665). The BALF smear and acid-fast staining were negative. On day 12, ESR and hsCRP did not significantly decrease, the patient's symptoms did not improve, and repeated chest CT showed no significant improvement (Figures 1A2, B2). Further inquiry of the patient revealed that he had regularly consumed “Shatangju” mandarin, a citrus fruit, in large quantities for the past 3 months. The patient was started on oral itraconazole capsules (200 mg each time, twice a day, taken immediately after meals) for antifungal therapy (day 12) without the above-mentioned antibiotics. Bronchoscopy (day 13) was performed again, and the BALF was collected for fungal and bacterial cultures. On day 16, *Penicillium* species were found in the fungal culture of the first BALF, and on day 19, the second BALF fungal culture also supported the growth of the same type of *Penicillium* species (Figure 3). The internal transcribed spacer (ITS) identification of the species was performed. The colony was identified as *P. digitatum*.

## An experimental method for *in vitro* antifungal susceptibility testing

1. The surface of bacterial colonies is rinsed with sterile physiological saline to create a spore suspension with a concentration of 0.5–1 McFarland.
2. A sterile swab is dipped into the bacterial suspension and squeezed to dry. Then, the agar surface is gently and evenly streaked in three directions, ensuring that the moisture is absorbed by the agar in <15 min.
3. An Etest strip is placed on the agar surface (ensuring the agar surface is dry and uniformly smooth before applying) and then incubated at 35°C for 48–72 h until clear inhibition zones appear.

## Result interpretation

The interpretation of results varies based on the different antimicrobial mechanisms of various drugs.

1. For amphotericin B, the MIC value is interpreted at the point where 100% of bacterial growth is inhibited.
2. For 5-fluorocytosine, the MIC value is interpreted at the point where 90% of bacterial growth is inhibited.
3. For azoles and echinocandins, the MIC value is interpreted at the point where 80% of bacterial growth is inhibited.

*In vitro* antifungal susceptibility testing by broth microdilution methods showed the following minimum inhibitory concentrations: amphotericin B at 2 μg/ml, voriconazole at 0.06 μg/ml, micafungin at ≤ 0.25 μg/ml, and itraconazole at 0.125 μg/ml. After antifungal therapy, the patient's ESR and hsCRP levels significantly decreased. The chest CT scan on day 18 showed improvement in pulmonary inflammation and pleural effusion compared to before (Figures 1A3, B3). The patient's infection was evaluated as being under control, and he was discharged to continue with outpatient treatment, with oral itraconazole (200 mg each time, twice a day).

After 15 days, considering the patient experienced mild gastrointestinal discomfort while the pulmonary condition was well controlled, the itraconazole dosage was reduced to 200 mg once daily for 1 month. A follow-up chest CT scan on 10 March 2023 showed further absorption of pulmonary lesions (Figures 1A4, B4). There has been no recurrence during the 1 year follow-up period. Although no further CT scans were performed during this time, the patient remained in good health with no worsening of respiratory symptoms.

## Discussion

*Penicillium* is a ubiquitous genus of mold, with many soil-borne saprophytic species, thriving in environments with moisture and decaying vegetation (8). In clinical samples, it is often isolated as a contaminant and overlooked, but it has become an opportunistic pathogen in immunocompromised patients. Except for *Talaromyces marneffei* (formerly known as *P. marneffei*), *Penicillium* infections are associated with allergic pneumonia and immunosuppressive diseases (9, 10) and, in severe cases, it can lead to systemic disseminated infections (11). *P. digitatum* is one of the most destructive pathogens of decaying citrus fruits, causing up to 90% of postharvest losses (12).

We searched for case reports and case series of pulmonary infections due to *Penicillium* species during 1995–2024 in PubMed using the keywords “(lung) AND (*Penicillium*),” except *Talaromyces marneffei*. Our search yielded 29 case reports that are summarized in Table 1. It summarizes data from 37 patients, of whom 15 (41%) were men and 13 (35%) were women. A total of 16 patients (43%) were over 50 years old, and 18 patients had their fungal species identified at the species level, with the following species being most common: *Penicillium citrinum* (4 patients, 11%), *P. digitatum* (2 patients, 5%), *Penicillium chrysogenum* (2 patients, 5%), *Penicillium brevicompactum* (2 patients, 5%), and *Penicillium purpurogenum* (2 patients, 5%). Regarding predisposing factors, 23 patients (62%) had occupational exposure, which mostly resulted in allergic pneumonia. Moreover, four patients (11%) had underlying pulmonary diseases, and seven patients (19%) were immunocompromised, including five (14%) with a history of cancer. In terms of treatment, 14 patients (38%) received steroid therapy, mainly for occupational exposure-related hypersensitivity pneumonitis. *Penicillium* infections were mostly treated with amphotericin B and itraconazole. Specifically, four patients (11%) were treated with amphotericin B, and nine patients (24%) received azole medications, with four out of these nine patients (4%) choosing itraconazole. Ultimately, 22 patients (59%) showed good recovery after treatment.

**Table 1:** Case reports and case series of *Penicillium* species pulmonary infection during 1995–2024 in PubMed (M, male; F, female; RW, recover well).

**References**	**Country/Age/gender**	**Pathogenic bacterium**	**Predisposing factors**	**Treatment**	**Outcome**
Cámara et al. (13)	NA	*Penicillium brevicompactum*	Allogeneic bone marrow transplant recipient	NA	NA
D'Antoni et al. (14)	Italy/57/M	*Penicillium chrysogenum*	Lung cancer	Itraconazole	RW
Nakagawa-Yoshida et al. (15)	Canadian/79,66,76/3 M	*Penicillium brevicompactum*, *Penicillium olivicolor*	Occupational exposure	Steroid therapy/prednisone	Died
Mok et al. (16)	China/60/F	*Penicillium citrinum*	Acute leukemia	Amphotericin B, itraconazole	Died
Bates et al. (17)	England/38/M	*Penicillium species*	Occupational exposure	Prednisolone	RW
Qadir and Cunha et al. (18)	America/67/M	*Penicillium peritonitis*	Peritoneal dialysis	Fluconazole	RW
Cormier et al. (19)	Canada/54/M	*Penicillium species*	Occupational exposure	Prednisolone	RW
Perry et al. (20)	NA	*Penicillium species*	Occupational exposure	Steroids	RW
Breton et al. (21)	NA	*Penicillium purpurogenum*	Hematology disease	NA	NA
Rivero et al. (22)	Mar del Plata/56/F	*Penicillium species*	Occupational exposure	NA	NA
Novotny and Dixit et al. (23)	Hispanic-Filipino/ <1/M	*Penicillium purpurogenum*, *Trichoderma species*	Occupational exposure	Methylprednisolone	RW
Ohnishi et al. (24)	Japan/37/F	*Penicillium corylophilum*	Occupational exposure	NA	NA
Lee et al. (25)	Korea/30/F	*Penicillium species*	Occupational exposure	Corticosteroid	RW
Yoshikawa et al. (26)	Japan/47/F	*Penicillium citrinum*	Occupational exposure	Methylprednisolone	RW
Laguna et al. (27)	Mexican/16/F	*Mycobacterium kansasii*, *Penicillium species*	Cystic fibrosis, abdominal pain	Amikacin, azithromycin, ethambutol, rifampin	NA
Guillot et al. (28)	France/27,49,38/3 NA	*Penicillium species*	Occupational exposure	Corticosteroid	RW
Amano et al. (29)	Japan/54/F	*Penicillium species*	Occupational exposure	NA	NA
Yasui et al. (30)	Japan/NA/3 NA	*Penicillium species*	Occupational exposure	NA	NA
Morell et al. (31)	Spain/avg. age 41/3 F	*Penicillium species*	Occupational exposure	NA	RW
Chien et al. (32)	Asian/57/F	*Penicillium species*	Franklin disease	Amphotericin B	RW
Geltner et al. (33)	Austria/56/M	*Penicillium chrysogenum*	Underwent left single lung transplant for α1-antitrypsin deficiency with severe lung emphysema	Posaconazole, caspofungin	Died
Chen et al. (34)	China/56/F	*Penicillium capsulatum*	Type 2 diabetes	Fluconazole, caspofungin	RW
Dillard and Ortega et al. (35)	American/44/M	*Cunninghamella species*, *Aspergillus fumigatus*, *Penicillium species*	History of pulmonary, tuberculosis	Itraconazole	RW
Oshikata et al. (2)	Japan/78/M	*Penicillium digitatum*	Bronchial asthma and pulmonary emphysema	Voriconazole, amphotericin B, fluconazole, itraconazole	RW
Shokouhi et al. (36)	Iran/44/M	*Penicillium notatum*, *Pneumocystis jiroveci*	Acute Myeloid Leukemia	Voriconazole, primaquine, clindamycin	RW
Zhao et al. (37)	China/67/M	*Penicillium citrinum*	Acute fibrinous and organizing pneumonitis, Type 2 diabetes	Methylprednisolone, amphotericin B, voriconazole	RW
Kutsuzaw et al. (38)	Japan/66/M	*Penicillium digitatum*	Occupational exposure, smoker	NA	RW
Beena et al. (39)	India/60/M	*Penicillium citrinum*	Multiple myeloma	NA	NA
Marruchella et al. (40)	Italy/42/F	*Penicillium species*	Occupational exposure	Corticosteroid therapy	RW

To the best of our knowledge, only two cases of pulmonary infection caused by *P. digitatum* have been reported (2, 3). Oshikata (2) reported the first case of pulmonary infection caused by *P. digitatum* in an elderly Japanese man who had emphysema and was undernourished. The second case was reported by Iturrieta-González et al. (3) in a late-term pregnant young woman who developed new nodular lesions in the lungs after being infected with SARS-CoV-2 and underwent termination of pregnancy, received treatment with corticosteroids, antibiotics, and mechanical ventilation. This infection was ultimately identified as *P. digitatum*.

The patient, in this case, had a clear history of consuming a large number of citrus fruits, including those with signs of mild rot, during the disease-onset period. There is also a possibility of inhaling spores of the *Penicillium* genus present in the air. Additionally, the patient was elderly and malnourished, had long-term smoking history and emphysema, had recently undergone major surgery, and was infected with COVID-19. The patient was in the recovery phase of COVID-19 infection, and his blood lymphocyte count remained below normal upon hospital admission. This indicates that the patient's cellular immune function has not yet fully recovered from the COVID-19 infection. These factors may have collectively contributed to the immunocompromise in the patient and facilitated the occurrence of lung infection due to *P. digitatum*.

Only individual case reports have documented successful treatment of *Penicillium* infections with itraconazole, amphotericin B, or fluconazole (4, 41). In the report by Oshikata et al. (2), the patient finally received a multidrug combination therapy of itraconazole, amphotericin B, and fluconazole, but the patient's condition did not improve. In the report by Iturrieta-González et al. (3), antifungal susceptibility testing showed *in vitro* activity of amphotericin B, voriconazole, and itraconazole, and the patient received itraconazole 400 mg/day, and the condition was effectively controlled after 10 days. The patient in our case also received itraconazole and showed a good therapeutic response. Currently, there are no relevant guidelines regarding the treatment of *Penicillium*. The preferred treatment involves administering Amphotericin B at a dosage of 0.5–1 mg/kg/day for 2 weeks, followed by maintenance therapy with Itraconazole at a dosage of 200 mg twice daily for 10 weeks (12–14). More research and reports are needed to explore the antifungal treatment regimens for Penicillium.

## Conclusion

To the best of our knowledge, this is the first reported case of pulmonary infection caused by *P. digitatum* in China and the third case reported worldwide. The patient was an elderly man, undernourished, had emphysema, was in the recovery period of COVID-19 infection and surgery, and had a history of exposure to citrus fruit pathogens, which may have contributed to the occurrence of lung infection caused by *P. digitatum*. The patient's pulmonary fungal infection was effectively controlled with the use of itraconazole antifungal therapy. Although human infection with *P. digitatum* is considered rare, as an emerging opportunistic pathogen, clinical attention is still needed when it is isolated from immunocompromised patients.

## Data availability statement

The datasets presented in this study can be found in online repositories. The names of the repository/repositories and accession number(s) can be found in the article/supplementary material.

## Ethics statement

Written informed consent was obtained from the individual(s) for the publication of any potentially identifiable images or data included in this article. Written informed consent was obtained from the participant for the publication of this case report.

## Author contributions

XS: Conceptualization, Writing – original draft. JY: Methodology, Writing – original draft. PL: Data curation, Writing – original draft. WG: Formal analysis, Writing – original draft. ZF: Supervision, Writing – review & editing. CZ: Writing – original draft. YH: Writing – original draft. YG: Formal analysis, Writing – original draft. LZ: Conceptualization, Supervision, Writing – review & editing.
